# Supplementary material for: Dynamic Nomogram for Predicting Long-Term Survival in Terms of Preoperative and Postoperative Radiotherapy Benefits for Advanced Gastric Cancer
Source: Int J Environ Res Public Health. 2023 Feb 3;20(3):2747. doi: 10.3390/ijerph20032747 (PMC9915292; doi:10.3390/ijerph20032747)
Supplement: Supplementary file 1 [file ijerph-20-02747-s001.zip › ijerph-2122011-supplementary.pdf]

Supplementary Materials:

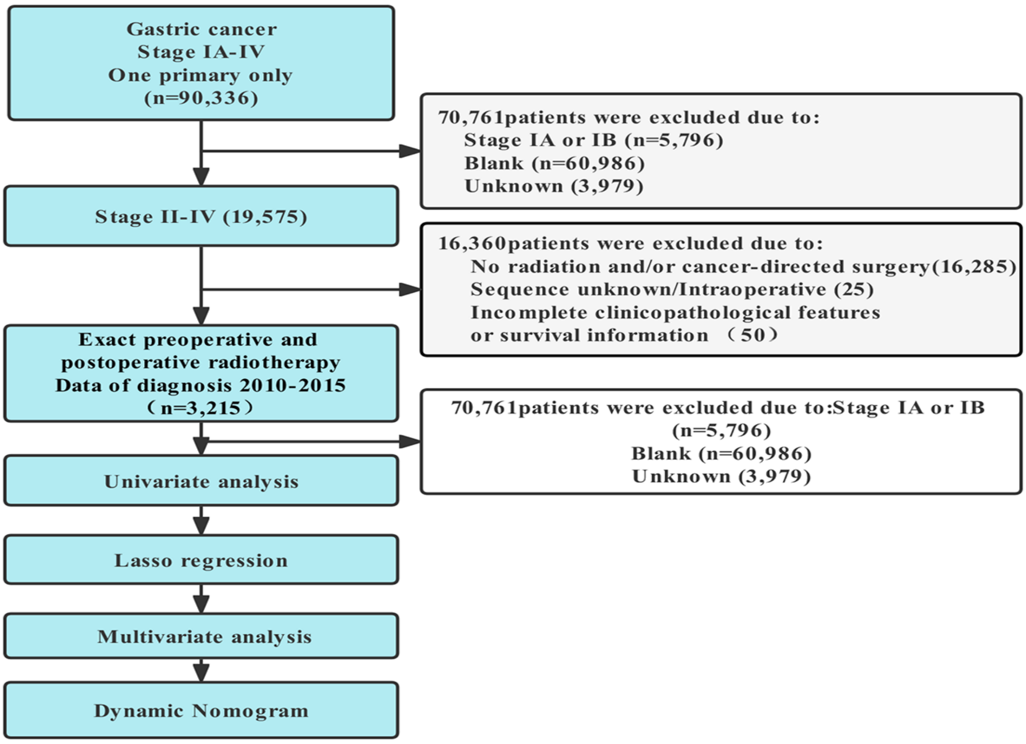

Supplementary figure1. Our research workflow chart.

Table S1. Univariate analysis of factors associated with mortality of patients with gastric cancer.

Characteristics	Total mortality (n=2124)	PERT (n=567)	P	PORT (n=982)	P	Pa
Age			0.009		0.034	<0.001
≤ 65	1213 (63.8)	460(63.4)		753(64.0)		0.478
> 65	911 (69.4)	332(69.5)		579(69.3)		0.574
Sex			0.102		0.663	0.629
Male	1511 (66.5)	669(66.8)		842(66.4)		0.652
Female	613 (64.9)	79(60.9)		490(66.0)		0.105
Race			0.281		<0.001	<0.001
White	1547 (67.9)	705(66.4)		842(69.2)		0.103
Black	251 (66.6)	33(65.9)		218(66.7)		0.730
Other	326 (58.2)	54(58.7)		272(58.1)		0.773
Marital status			<0.001		0.263	0.006
Married	1352 (64.5)	520(63.3)		832(65.3)		0.348
Divorced/Separated	224 (71.6)	89(69.5)		135(73.0)		0.650
Single	300 (65.2)	115(69.3)		185(62.9)		0.432
Widowed	151 (74.0)	42(85.7)		109(70.3)		0.006
Unknown	97 (68.8)	26(66.7)		71(69.6)		0.927
Primary site			<0.001		<0.001	<0.001
Cardiac/fundus	1063 (66.5)	712 (64.6)		351 (64.4)		<0.001
Body	139 (63.5)	12 (80.0)		127 (62.3)		0.045
Antrum/pylorus	431 (61.3)	12 (75.0)		419 (61.0)		0.305
Lesser/greater curvature	208 (58.4)	20 (66.7)		188 (57.7)		0.207
Other	283 (78.2)	36 (90.0)		247 (76.7)		0.002

Histology			0.125		0.760	0.353
Adenocarcinoma	1911 (66.0)	754 (65.5)		1157 (66.3)		
Other	213 (66.8)	38 (71.7)		175 (65.8)		
TNM Stage			<0.001		<0.001	<0.001
II	511 (50.7)	211(54.0)		300(48.7)		0.018
III	1908 (70.0)	514(69.3)		821(70.4)		0.674
IV	278 (92.7)	37(94.4)		211(92.1)		0.190
T stage			<0.001		<0.001	<0.001
T1-2	262 (54.8)	106(59.6)		156(52.0)		0.070
T3	1186 (63.9)	604(65.2)		582(62.5)		0.131
T4	617 (75.5)	77(81.1)		540(64.8)		0.019
Tx	59 (93.7)	5(100.0)		54(93.1)		0.483
N stage			<0.001		<0.001	<0.001
N0	353 (56.7)	154(54.2)		199(58.7)		0.249
N1	682 (61.7)	356(62.5)		326(60.9)		0.796
N2	533 (69.0)	211(79.6)		322(63.4)		<0.001
N3	556 (77.9)	71(83.5)		485(77.1)		0.025
M stage			<0.001		<0.001	<0.001
M0	1846 (63.3)	725(64.0)		1121(62.9)		0.259
M1	278 (92.7)	67(94.4)		211(92.1)		0.190
Differentiation			<0.001		<0.001	<0.001
Poorly	1391 (68.2)	454(71.2)		937(66.8)		0.073
Moderately	470 (60.0)	224(58.6)		246(81.3)		0.719
Well	44 (53.7)	25(58.1)		19(48.7)		0.139
Undifferentiated	219 (70.9)	89(63.1)		130(77.4)		0.003
Summary stage			<0.001		<0.001	<0.001
Localized	125 (51.9)	61(56.5)		64(48.1)		0.092
Regional	1583 (63.6)	578(64.2)		1005(63.2)		0.348
Distant	416 (86.0)	153(78.1)		263(91.3)		<0.001
Lauren type			0.245		0.004	0.006
Intestinal	199 (59.8)	36(60.0)		163(59.7)		0.526
Diffuse	160 (63.5)	20(71.4)		140(62.5)		0.396
Mixed	69 (69.0)	16(76.2)		53(67.1)		0.179
Unknown	1696 (67.0)	720(65.8)		976(68.0)		0.120
Tumor size			0.511		<0.001	<0.001
≤3 cm	416 (57.3)	202(63.1)		214(52.7)		<0.001
3-5 cm	596 (64.9)	230(66.3)		366(64.0)		0.350
>5 cm	770 (69.2)	193(67.0)		577(69.9)		0.483
Unknown	342 (74.8)	167(67.1)		175(84.1)		<0.001
Bone metastases			0.700		0.545	0.479
Yes	88 (68.2)	38 (67.9)		50 (68.5)		0.811
No/Unknown	2036 (66.0)	754 (65.7)		1282 (66.2)		0.778
Brain metastases			0.607		<0.001	<0.001
Yes	35 (89.7)	3 (75.0)		32 (91.4)		0.127
No/Unknown	2089 (65.8)	789 (65.7)		1300 (65.8)		0.917
liver metastases			<0.001		<0.001	<0.001
Yes	69 (95.8)	21 (100.0)		48 (94.1)		0.704
No/Unknown	2055 (65.4)	771 (65.2)		1284 (65.5)		0.891
lung metastases			<0.001		<0.001	<0001
Yes	29 (93.5)	11(100.0)		18(90.0)		0.752
No/Unknown	2095 (65.8)	781(65.5)		1314(66.0)		0.935
Chemotherapy			0.588		<0.001	<0.001

Yes	2000 (65.3)	785(65.7)	1215(65.0)	0.405
No/Unknown	124 (80.5)	7(63.6)	117(81.8)	0.612

Abbreviations: PERT, Preoperative radiotherapy; PORT, Postoperative radiotherapy. P for intra-group univariate survival analysis; Pa for inter-group univariate survival analysis.

Table S2. The Overall Survival of patients in the preoperative radiotherapy and postoperative radiotherapy groups (Stage-stratified survival analysis).

OS	stage II			stage III			stage IV		
	PERT	PORT	P	PERT	PORT	P	PERT	PORT	P
1-Year	86.4	89.9	0.018	81.1	80.0	0.668	54.9	41.3	0.182
3-Year	57.1	63.8	0.018	42.4	40.0	0.567	19.7	11.8	0.163
5-Year	44.3	53.8	0.001	29.9	29.8	0.112	15.5	5.2	0.209
Total	45.9	50.7	0.018	30.4	28.9	0.674	5.6	6.6	0.190

Abbreviation: OS, overall survival, PERT, preoperative radiotherapy; PORT, postoperative radiotherapy.
